# MitraNav: Design and Modeling of a Robotic System for Transseptal Mitral Valve Replacement with Dock Catheter Task-Space Control

**DOI:** 10.1007/s10439-026-04154-1

**Published:** 2026-05-02

**Authors:** Weizhao Wang, Zhenguan Tu, Jiyu Cai, Shuangyi Wang, Richard J. Housden, Kawal Rhode

**Affiliations:** 1 Department of Surgical & Interventional Engineering, King’s College London, Strand, London, WC2R 2LS U.K.; 2 Institute of Automation, Chinese Academy of Sciences, 95 Zhongguancun East Road, Beijing, 100190 China; 3 School of Artificial Intelligence, University of Chinese Academy of Sciences, No. 1 Yanqihu East Road, Beijing, 101408 China; 4 Centre for Artificial Intelligence and Robotics Hong Kong Institute of Science & Innovation, Chinese Academy of Sciences, Science Park West Avenue, Hong Kong, China

**Keywords:** Transcatheter mitral valve replacement, Dock catheter, Medical robot, Multi-segment continuum robot, Task-space control

## Abstract

****Purpose**:**

Transcatheter mitral valve replacement (TMVR) is a promising therapy for reducing mitral regurgitation in select patients. Direct tip control of multi-segment catheters can simplify manipulation and enable precise, stable positioning. This paper introduces MitraNav, the first robotic system developed for dock-based TMVR, focusing on system design and task-space control of the dock catheter.

****Methods**:**

Built on a previously validated guide-sheath robot, MitraNav is upgraded to support concentric multi-catheter manipulation via a unified actuation mechanism. It suits both dock and balloon catheters, enabling all catheter manipulation using a single robotic system. A two-segment bending model maps joint space to task space through an intermediate configuration space. A two-stage control strategy is then applied: joint-space control allows for rapid approach to the mitral annulus, followed by task-space control for direct tip positioning. To improve accuracy and handle joint limits, the task-space controller incorporates Jacobian normalization to balance joint contributions, null-space optimization to regulate joint motion without affecting the primary task, and a PD controller to reduce tracking errors.

****Results**:**

Trajectory-following experiments demonstrate feasibility of direct tip control, with maximum errors of 2.38 mm, 2.53 mm, and 2.46 mm in three trials. These positional errors are within the range of acceptable accuracy commonly considered for catheter-based cardiac interventions.

****Conclusion**:**

MitraNav represents the foundation for intuitive catheter manipulation and offers the potential for semi-autonomous robotic dock-based TMVR.

## Introduction

**Fig. 1:**
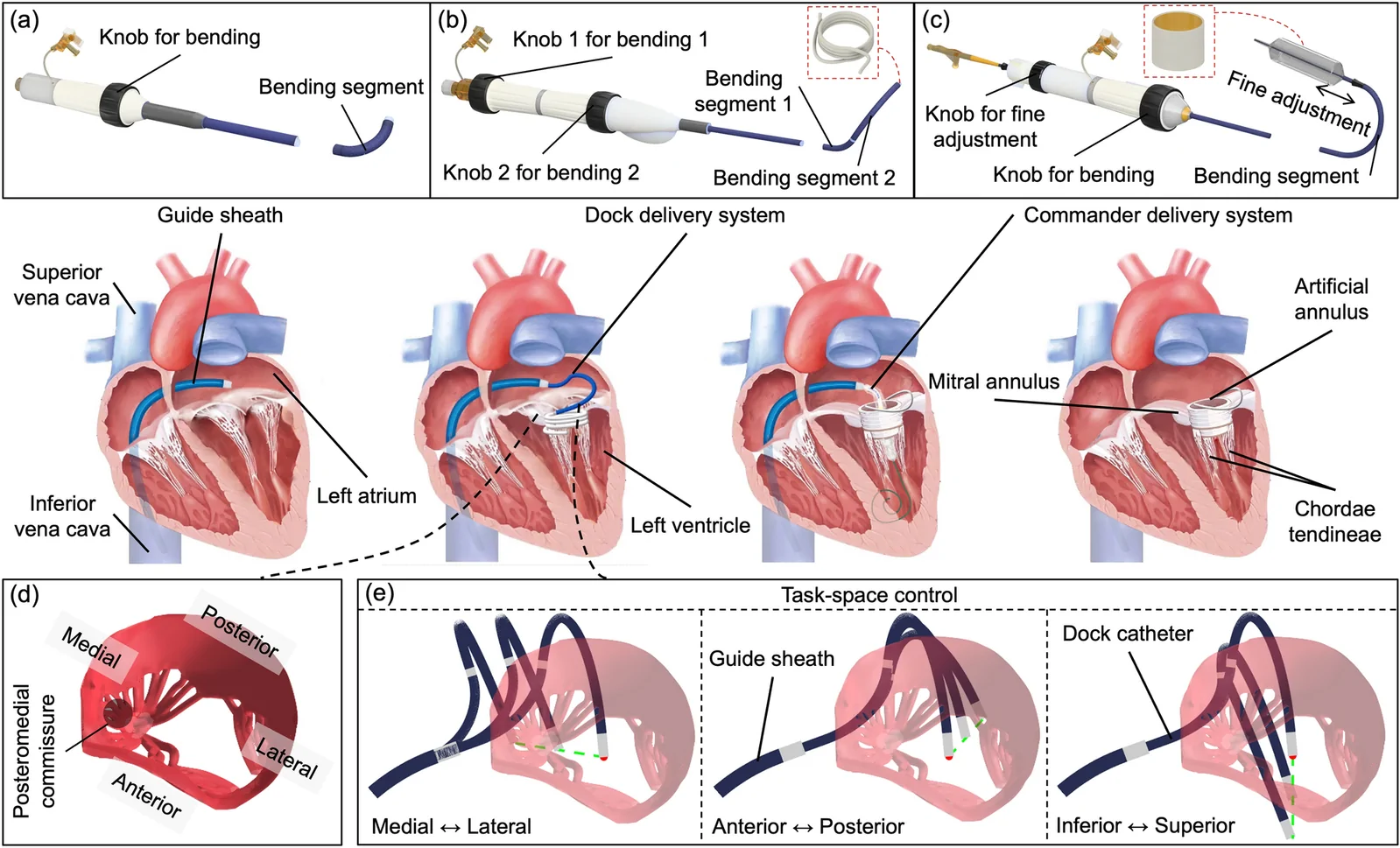
Dock-based TMVR procedure using the SAPIEN M3 delivery system:** a** The cardiac guide sheath establishes a delivery pathway from the femoral vein to the left atrium.** b** The dock delivery system is guided into the left ventricle and deploys a nitinol dock to encircle the chordae tendineae.** c** The Commander delivery system delivers a prosthetic valve and implants it within the dock.** d** The target docking location is beneath the posteromedial mitral commissure.** e** Task-space control could enable intuitive and fine adjustment along anatomical directions

Transcatheter techniques have significantly advanced the treatment of valvular heart disease [1, 2]. Among these, transcatheter edge-to-edge repair (TEER) has become an established therapy for mitral regurgitation (MR) [3, 4], one of the most common valvular heart diseases worldwide [5, 6]. However, TEER is not suitable for all patients, especially those with complex valve anatomy or severe leaflet pathology. For these cases, transcatheter mitral valve replacement (TMVR) has emerged as a promising alternative. Growing evidence suggests that TMVR may offer more consistent and complete MR reduction in select patients [7–10].

Unlike the relatively rigid and circular aortic annulus, the mitral valve (MV) has a dynamic, saddle-shaped annulus that changes throughout the cardiac cycle [11, 12]. Furthermore, mitral annular calcification often exhibits an irregular and non-circumferential distribution, frequently extending toward the mitral leaflet insertion and, in some cases, into the valve orifice. This anatomical variability may limit reliable prosthesis anchoring during TMVR and can increase the risk of complications such as device migration and paravalvular leakage [13, 14]. To address these challenges, some TMVR systems use docking mechanisms that encircle the chordae tendineae below the mitral annulus. This technique effectively creates an “artificial annulus,” providing a stable landing zone for valve anchoring (“valve-in-ring” approach) [15]. As shown in Fig. 1, the SAPIEN M3 system (Edwards Lifesciences Inc., Irvine, CA, USA) is an example of this approach and is currently the first and only approved transseptal TMVR system [16]. The procedure can be simplified into three key steps [17, 18]: 1) A transseptal deflectable guide sheath (Fig. 1a) is advanced into the left atrium to establish a delivery pathway from the femoral vein; 2) A dock delivery system (referred to as the dock catheter hereafter, shown in Fig. 1b) is inserted through the sheath and used to deploy a self-expanding nitinol dock into the left ventricle; 3) The Commander delivery system (Fig. 1c), a balloon catheter, then replaces the dock catheter and delivers the balloon-expandable prosthetic valve within the dock during rapid ventricular pacing. In our previous work [19], we investigated the safe delivery of the sheath. In the present study, we focus on the subsequent dock delivery step, which is also recognized as technically challenging [20]. The dock catheter has two bending segments with a $90^\circ$ offset and is manually actuated via two knobs on the handle (Fig. 1b). While this multi-segment continuum design provides the flexibility needed to navigate the intricate mitral anatomy [20], it also makes precise tip placement more difficult. Clinically, the recommended target location for the dock tip is just beneath the posteromedial mitral commissure (Fig. 1d), defined as the junction of the anterior and posterior leaflets at the medial (toward the interatrial septum) and posterior aspect of the annulus. Additionally, the catheter tip must be positioned beneath the anterior mitral leaflet to facilitate forward advancement of the dock [17]. Coordinating multiple coupled degrees of freedom (DoFs) using direct joint manipulation is unintuitive and imposes a high cognitive load on the operator, making accurate tip placement technically demanding. To address this challenge, task-space control of the dock catheter via a robotic system provides a more intuitive paradigm. By mapping user input directly to the catheter tip motion in Cartesian space (Fig. 1e), this approach could reduce control complexity and enable fine, stable positioning in clinically relevant target regions.

Robotic systems have been proposed to enhance catheter manipulation by improving precision, enabling remote operation, and reducing radiation exposure for cardiologists [21–26]. Recent reviews on current and future applications of robotics in structural heart interventions highlight the rapid growth in this field and the increasing attention toward robotic technologies [27, 28]. In mitral interventions, the first-in-human robotic-assisted mitral TEER under pure echocardiographic guidance was reported using the Kyrin TEER system (Shenqi Medical, Shanghai, China) in 2024 [29]. In this procedure, the transseptal puncture was performed manually, while all subsequent steps (including clip delivery) were completed using a newly developed robotic system from SurgiPulse Robotics (Shanghai, China). In 2025, Capstan Medical (Santa Cruz, California, USA) achieved the first-in-human robotic-assisted TMVR, using a customized MV prosthesis and a dedicated robotic platform [30]. This system enables the operator to gradually expand and reposition the valve for optimal alignment. Despite these milestones, available technical details indicate that existing systems still rely on joint-level control and require highly experienced operators. Enabling task-space tip control could reduce operator dependency, lower the learning curve for less-experienced users, and serve as a foundation for future semi-autonomous or autonomous procedures. Nayar and Qi et al.[31–33] developed a robotic transcatheter delivery system for MV implantation, incorporating a constant curvature kinematic model and a task-space controller to achieve accurate tip positioning. Zhang and Bicchi et al.[34, 35] proposed a 3 DoFs robotic platform for MitraClip$^\textrm{TM}$ manipulation, employing a task-space path planner and a PID-based control architecture to guide the device tip. These studies demonstrate the increasing application of task-space control in robotic mitral interventions. However, to the best of our knowledge, no robotic system has been specifically developed for dock-based TMVR. The unique anatomical and mechanical challenges involved in navigating and positioning a multi-segment docking catheter, particularly within the left atrium and toward the mitral annulus, remain unaddressed by existing robotic platforms.

In this paper, we propose MitraNav, the first robotic system specifically developed for transseptal, dock-based TMVR. Its design, modeling, and task-space control of the dock catheter are presented. MitraNav builds on a previously developed robotic platform for cardiac guide-sheath manipulation [19], now upgraded to support concentric, multi-catheter operation. A newly designed unified catheter platform with flexible clamping and actuation configurations enables manipulation of both the dock and balloon catheters. This could make the system capable of performing the latter two steps of the TMVR procedure. We introduce a two-segment bending model that maps joint space to task space through an intermediate configuration space. To address actuator nonlinearities such as hysteresis and dead zones, we integrate a previously validated compensation model [36], and combine the kinematics of both bending segments to estimate the catheter tip position. A two-stage controller is implemented: joint-space control enables rapid approach to the mitral annulus, while task-space control provides direct tip positioning. To improve accuracy and handle joint limits, the task-space controller incorporates Jacobian normalization to balance joint contributions, null-space optimization to regulate joint motion without affecting the primary task, and a PD controller to reduce tracking errors. Finally, the system was validated by executing a predefined figure-eight trajectory with both straight and curved segments, starting from the center and oriented to test three-dimensional catheter tip positioning.

The remainder of this article is organized as follows. Section Materials and Methods presents the detailed design of the proposed MitraNav system for the TMVR procedure, followed by the modeling and task-space control of the dock catheter, and the experimental setup for validation. Section Results describes the validation results. Finally, Section Discussion discusses the system’s performance and limitations, summarizes the conclusions, and outlines future work, including potential improvements and plans for clinical validation.

## Materials and Methods

### Robot Design and Fabrication

This section describes the design, fabrication, and teleoperation of the MitraNav system.

**Fig. 2:**
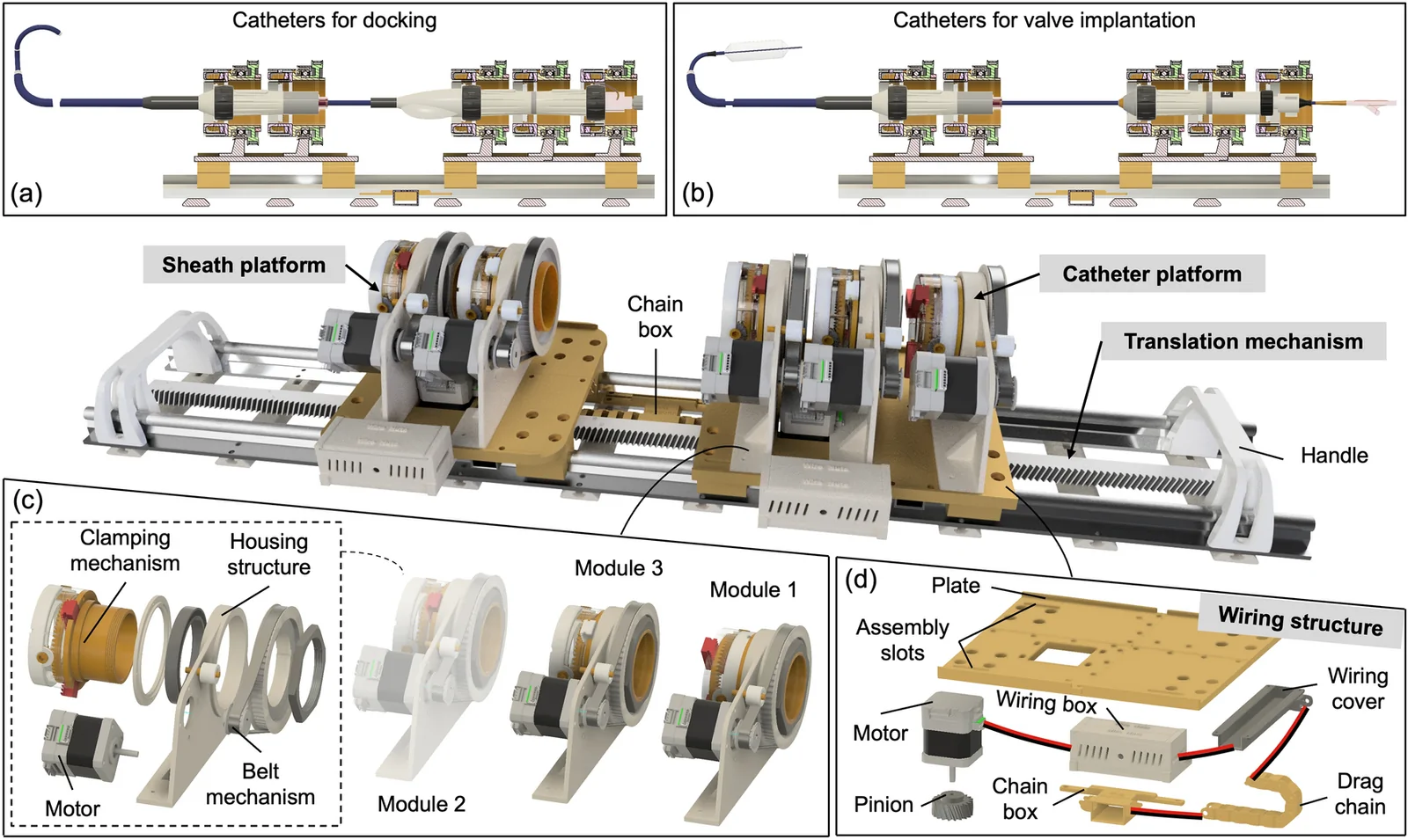
The robotic system is upgraded with a catheter platform and a wiring structure, in addition to the existing sheath platform and translation mechanism:** a** and** b** show the adaptability of the catheter platform, which can accommodate both a dock catheter and a balloon catheter by adjusting the clamping distance between the knobs and the handle. It also enables simultaneous concentric control of the sheath and catheter.** c** The catheter platform includes three actuation modules that work together to achieve independent handle rotation and knob turning.** d** The wiring structure organizes the motor cables and routes them safely outside the robot to protect the wiring during movement

#### Robot Design

Previously, we developed a robotic system for cardiac guide-sheath manipulation and demonstrated its safe delivery through the fossa ovalis [19]. In this paper, we shift focus to the manipulation of the following functional catheter. The system has been upgraded with a new catheter platform that enables independent control of an additional catheter, along with a redesigned wiring structure for signal and power management. These additions are built on the existing sheath platform and translation mechanism, as shown in Fig. 2.

To enable simultaneous manipulation of the sheath and catheter, a new catheter platform was added behind the sheath platform. This configuration allows the catheter to be inserted concentrically into the guide sheath. The design supports manipulation of both the dock and balloon catheters using the same platform structure. Clamping configurations for docking and valve implantation are shown in Fig. 2a and 2b. The dock catheter includes two knobs, requiring three actuation modules for full control, as illustrated in Fig. 2c. Specifically, Module 1 actuates Knob 1, Module 2 controls Knob 2, and Module 3 stabilizes the catheter handle. Module 3 also manages catheter rotation. The relative motions of Modules 1 and 2 with respect to Module 3 regulate the knobs, thereby adjusting the two bending angles. Each module consists of a belt mechanism, a clamping mechanism, a motor, and a housing structure [36]. For the balloon catheter, assembly slots on the platform plate (Fig. 2d) allow Modules 1 and 2 to be repositioned. This adjustability allows customization of the clamping distance between the knobs and the handle, ensuring that the balloon catheter can also be securely held using the same platform.

With the gear rack mechanism, the catheter platform can be translated using only a motor and a pinion gear (Fig. 2d). The platform provides four DoFs for catheter manipulation as a whole. The plate measures 23.5 cm in length, and the catheter’s translation range exceeds 50 cm. This range is sufficient to meet anatomical requirements, as the estimated distance from the femoral vein puncture site to the fossa ovalis is approximately 40 to 50 cm [37]. Motor wires are converted to CAN bus signals within the wiring box and routed beneath the platform through a wiring cover, which connects to a drag chain. This structure protects the cables during catheter platform movement. The drag chain is then connected to a chain box, through which the wires exit the robotic system. For easier transport, two side-mounted handles are reinforced with structural ribs, improving both rigidity and grip during handling.

#### Robot Fabrication

To balance lightweight construction with adequate strength in critical transmission components, the pinions, jaws, and scroll plates of the actuation module, as well as the gears and racks for translational motion, were fabricated from nylon by Shenzhen Jinma 3D Technology Co., Ltd. All remaining components were 3D printed using PLA filament (Shenzhen Esun Industrial Co., Ltd.) on an Original Prusa MINI+ printer (Prusa Research a.s., Czech Republic). Parts were designed in CAD software (Fusion 360, Autodesk Inc., USA) and converted into 3D-printable files using PrusaSlicer (version 2.6.1, Prusa Research a.s., Czech Republic). Linear motion was facilitated by two SBR12 guide shafts (1000 mm) and eight SBR12UU linear bearings, purchased from VEVOR via Amazon UK. Rotational transmission employed five timing belts sourced from a supplier in China. The actuation system was driven by NEMA 17 stepper motors (0.4 Nm torque, $1.8^\circ$ step angle, 42.3 × 42.3 mm frame, 5 mm shaft). Power was supplied by a 24 V, 300 W, and 12.5 A DC adapter (Shenzhen TaiFangsong Technology Co., Ltd.), and communication with the controller was enabled via a CAN-to-USB adapter from Shenzhen DSD Technology Co., Ltd.

#### Software Platform

A PC running Ubuntu 20.04 serves as the main controller. A software platform was developed in C++23 to enable remote joint-space manipulation of the robotic system. It processes input commands from a PlayStation 5 DualSense wireless controller (PS5, Sony, Minato, Tokyo, Japan) and transmits control signals to the actuation system via a CAN bus operating at 250 Kbps. Each joint is mapped to a dedicated button on the controller, and the control loop runs at 2 ms to ensure responsive input capture. For teleoperation, the CAN bus cable was extended to 25 m, allowing the robotic system to operate in the interventional room while the cardiologist remains in the control room. The software architecture is designed to be modular and hardware-independent, allowing the same joint-space control framework to be applied to different catheter pairs. A task-space control interface is also reserved, with its implementation described in Section Experimental Setup.

### Model Analysis

**Fig. 3:**
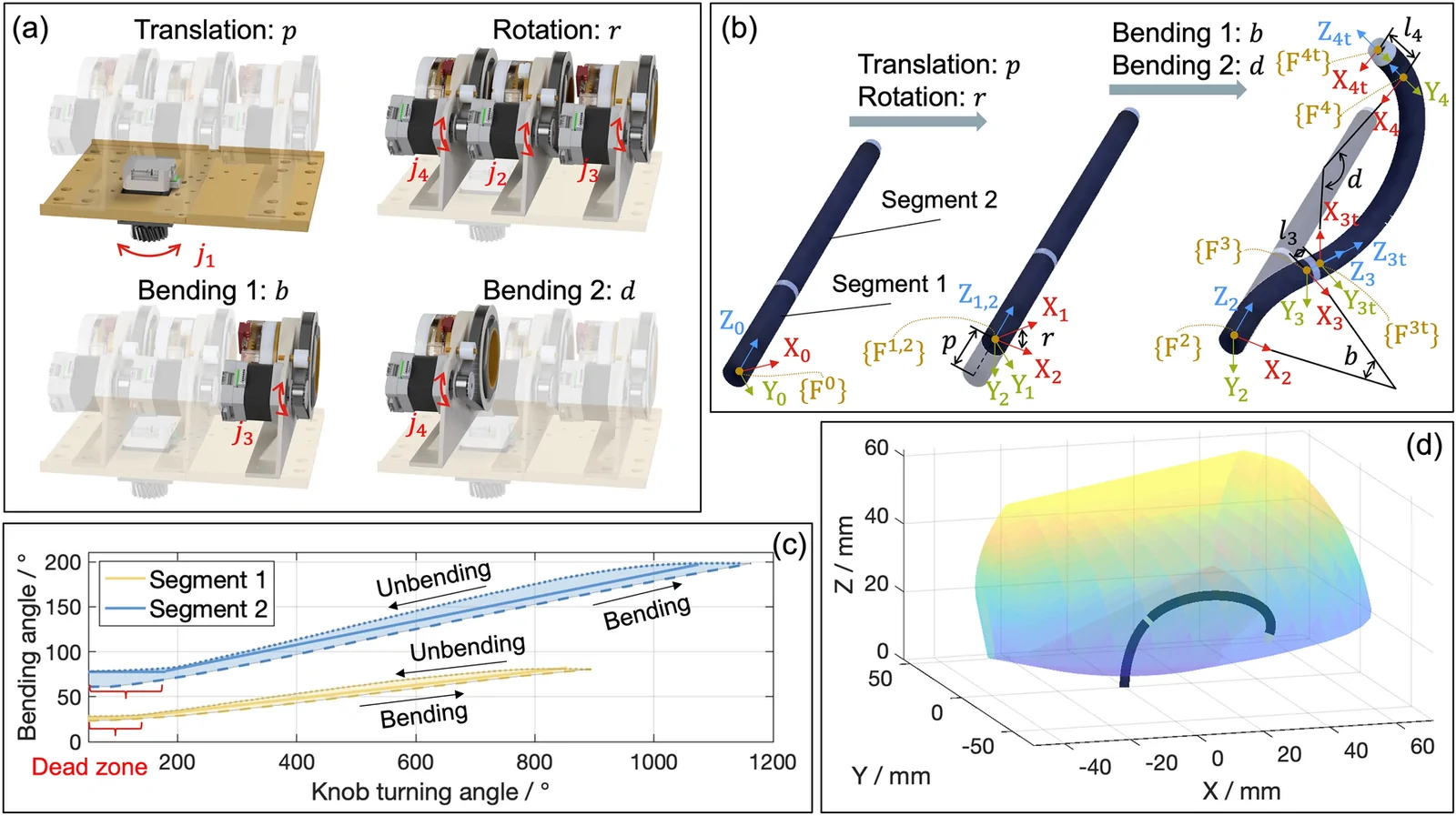
Model analysis of the dock catheter from joint space to task space: (a) Mapping from joint space $\left[ j_1, j_2, j_3, j_4\right] ^\textrm{T}$ to configuration space $\left[ p, r, b, d\right] ^\textrm{T}$. (b) Mapping from configuration space to task space ($\pmb {P}$). The coordinate systems are defined as base $\{\textrm{F}^0\}$, translation in $\{\textrm{F}^1\}$, rotation in $\{\textrm{F}^2\}$, first bending in $\{\textrm{F}^3\}$, and second bending in $\{\textrm{F}^4\}$. (c) Relationship between knob turning angle and bending angle reveals intrinsic nonlinear effects such as dead zone and hysteresis. A model  [36], combining a constant term (permanent deformation) and a linear term (active bending), is used to capture this behavior (solid lines). (d) Workspace of the robot-controlled dock catheter.

Given this structural complexity, we implemented a two-stage kinematic model to describe the behavior of the dock catheter from actuator input to tip pose (Fig. 3). The model consists of two components: 1) Joint space to configuration space mapping (Fig. 3a), which describes how internal catheter articulation responds to operator input, and 2) Configuration space to task-space mapping (Fig. 3b), which estimates the catheter tip’s position and orientation based on its internal shape.

In the joint space, the input variables are defined as motor turning angles ($j_1$ to $j_4$), corresponding to the catheter handle’s translation, rotation, and the turning of Knob 1 and Knob 2, respectively. These joint-level inputs are mapped to the configuration space, which includes the catheter tip’s translation (*p*), rotation (*r*), and the bending angles of Segment 1 (*b*) and Segment 2 (*d*). Translation and rotation are transmitted directly along the catheter shaft. They are scaled by the gear rack and belt transmission ratios, $\textrm{N}_1$ and $\textrm{N}_2$. Because the knobs are integrated into the rotating handle, the overall catheter rotation (*r*) must compensate for the rotational contributions of $j_3$ and $j_4$. The relationship between knob rotation and bending is nonlinear, primarily due to dead zones and hysteresis, as illustrated in Fig. 3c. A model combining a constant term and a linear function is employed to describe the bending behavior. Specifically, it captures both the constant offset (permanent bending deformation) and the linear relationship between the bending angle and the knob turning angle (active bending deformation), as illustrated by the solid lines in Fig. 3c. Following the modeling approach in [36], these relationships are expressed as $b = f_1(\textrm{N}_2 j_3)$ and $d = f_2(\textrm{N}_2 j_4)$. Further mathematical details can be found in [36]. The complete joint-to-configuration mapping is summarized as

1 $$\begin{aligned} \begin{bmatrix} p \\ r \\ b \\ d \end{bmatrix} = \begin{bmatrix} \textrm{N}_1& 0& 0& 0\\ 0& \textrm{N}_2& \textrm{N}_2& \textrm{N}_2\\ 0& 0& f_1(\textrm{N}_2j_3)/j_3& \\ 0& 0& 0& f_2(\textrm{N}_2j_4)/j_4 \end{bmatrix} \begin{bmatrix} j_1 \\ j_2 \\ j_3 \\ j_4 \end{bmatrix}. \end{aligned}$$

In task space, the configuration is defined using a series of coordinate frames from $\{\textrm{F}^0\}$ to $\{\textrm{F}^\textrm{4t}\}$, as shown in Fig. 3b. The base frame of the dock catheter is denoted as $\{\textrm{F}^0\}$, while frames $\{\textrm{F}^1\}$ to $\{\textrm{F}^4\}$ represent each DoF. The offsets along the Z-axis after the first and second bending segments are denoted by $l_3$ and $l_4$, respectively, with additional frames $\{\textrm{F}^\textrm{3t}\}$ and $\{\textrm{F}^\textrm{4t}\}$ accounting for these displacements. A $90^\circ$ rotation about the Z-axis between the two bending segments is represented by the transformation from $\{\textrm{F}^3\}$ to $\{\textrm{F}^\textrm{3t}\}$.

To formulate the kinematic model, we first construct the transformation matrix for the prismatic joint *p*. Next, we derive the transformation matrix for the rotational joint with rotation angle *r*. The resulting transformation matrix $_{0}^{2}\pmb {T}$ is given as

2 $$\begin{aligned} _{0}^{2}\pmb {T}(p,r) = \begin{bmatrix} \textrm{C}_r& -\textrm{S}_r& 0& 0\\ \textrm{S}_r& \textrm{C}_r& 0& 0\\ 0& 0& 1& p\\ 0& 0& 0& 0 \end{bmatrix} \end{aligned},$$

where $\textrm{S}_*$ and $\textrm{C}_*$ represent sin(*) and cos(*), respectively, with the subscript indicating the angle variable.

Common catheter bending models include the pseudo-rigid body (PRB) model, constant curvature (CC) model, and Cosserat rod model [38]. Each has its advantages and limitations, depending on the catheter type and operational requirements. In comparison, our previously proposed model [36], which accounts for elongation-induced deformation, has been shown to improve modeling accuracy for valve-related catheters. In our prior work, a random planar bending test reported mean errors of 1.90 mm, 1.84 mm, 0.59 mm, and 0.49 mm for the PRB, CC, Euler spiral, and the proposed model, respectively. Based on this model, the 2D tip positions are given by (*x*(*b*), *y*(*b*)) for Bending 1 and (*x*(*d*), *y*(*d*)) for Bending 2. The transformation matrix from $\{\textrm{F}^{2}\}$ to $\{\textrm{F}^\textrm{4t}\}$ is shown as

3 $$\begin{aligned} _{2}^\textrm{4t}\pmb {T}(b,d) = \begin{bmatrix} \textrm{C}_b& 0& \textrm{S}_b& x(b) + l_3\textrm{S}_b\\ 0& 1& 0& 0\\ -\textrm{S}_b& 0& \textrm{C}_b& y(b) + l_3\textrm{C}_b\\ 0& 0& 0& 1 \end{bmatrix} \begin{bmatrix} 0 & 1 & 0 & 0\\ -1 & 0 & 0 & 0\\ 0 & 0 & 1 & 0\\ 0 & 0 & 0 & 1 \end{bmatrix} \begin{bmatrix} \textrm{C}_d& 0& \textrm{S}_d& x(d) + l_4\textrm{S}_d\\ 0& 1& 0& 0\\ -\textrm{S}_d& 0& \textrm{C}_d& y(d) + l_4\textrm{C}_d\\ 0& 0& 0& 1 \end{bmatrix}. \end{aligned}$$

Finally, by multiplying matrices from (2) to (3), we can obtain a forward kinematic model (4) of the catheter tip relative to the robot base,

4 $$\begin{aligned} _{0}^\textrm{4t}\pmb {T}(p,r,b,d) = _{0}^{2}\pmb {T}(p,r)_{2}^\textrm{4t}\pmb {T}(b,d) = \left[ \begin{array}{c|c} \pmb {R} & \pmb {P} \\ \hline {\textbf {0}}_{1\times 3} & 1 \end{array} \right] \end{aligned}$$

5 $$\begin{aligned} \pmb {P} = \begin{bmatrix} p_\textrm{x} \\ p_\textrm{y} \\ p_\textrm{z} \end{bmatrix}= \begin{bmatrix} l_4(\textrm{S}_d\textrm{S}_r + \textrm{C}_d\textrm{C}_r\textrm{S}_b) + \textrm{C}_rx(b) + \textrm{S}_rx(d) + \textrm{C}_rl_3\textrm{S}_b + \textrm{C}_r\textrm{S}_by(d)\\ \textrm{S}_rx(b) - \textrm{C}_rx(d) - l_4(\textrm{C}_r\textrm{S}_d - \textrm{C}_d\textrm{S}_b\textrm{S}_r) + l_3\textrm{S}_b\textrm{S}_r + \textrm{S}_b\textrm{S}_ry(d)\\ p + y(b) + \textrm{C}_bl_3 + \textrm{C}_by(d) + \textrm{C}_b\textrm{C}_dl_4\\ \end{bmatrix} \end{aligned}$$

6 $$\begin{aligned} \pmb {R} = \begin{bmatrix} \textrm{C}_d\textrm{S}_r - \textrm{C}_r\textrm{S}_b\textrm{S}_d& \textrm{C}_b\textrm{C}_r& \textrm{S}_d\textrm{S}_r + \textrm{C}_d\textrm{C}_r\textrm{S}_b\\ -\textrm{C}_d\textrm{C}_r - \textrm{S}_b\textrm{S}_d\textrm{S}_r& \textrm{C}_b\textrm{S}_r& \textrm{C}_d\textrm{S}_b\textrm{S}_r - \textrm{C}_r\textrm{S}_d\\ -\textrm{C}_b\textrm{S}_d& -\textrm{S}_b& \textrm{C}_b\textrm{C}_d\\ \end{bmatrix} \end{aligned},$$

where $\pmb {P} \in \mathbb {R}^{3\times 1}$ and $\pmb {R} \in \mathbb {R}^{3\times 3}$ represent the position and orientation of $\{\textrm{F}^\textrm{4t}\}$ with respect to $\{\textrm{F}^{0}\}$. By combining the two mappings, (1) into (5) and (1) into (6), we obtain the complete kinematic relationship from the joint space to the task space of the dock catheter.

From Fig. 3c, the bending angle ranges for Segment 1 and 2 of the catheter are identified as $28^\circ -80^\circ$ and $78^\circ -198^\circ$, respectively. Using these parameters, along with a translation range of 0-20 mm and a rotation range of $-90^\circ$ to $90^\circ$, we computed the reachable workspace of the robotic catheter system based on the kinematic model. The workspace origin is defined at the distal tip of the guide sheath, as the dock catheter is deployed from within the sheath. The resulting workspace, shown in Fig. 3d, represents all achievable catheter tip positions within the system’s joint and bending limits. It forms a dome-shaped volume extending approximately 60 mm along the axial (Z) direction and ±50 mm in the lateral (X-Y) plane. This envelope reflects the spatial reach enabled by varying the joint inputs within their physical constraints. The curved trajectory embedded in the figure shows a representative shape of the catheter tip. Importantly, the workspace could align with the anatomical dimensions needed for MV docking via the transseptal approach. In large cohorts of patients undergoing percutaneous mitral interventions, left atrial diameters typically span 40–66 mm, with upper tertile values reaching  60 mm [39]. Given our workspace extends approximately 60 mm axially and ±50 mm laterally, it could support reliable catheter access, alignment, and docking at the mitral annulus within the left atrium.

The overall kinematics of the combined guide sheath and docking catheter can be obtained by composing the guide-sheath transformation matrix $_\textrm{s}^\textrm{0}\pmb {T}$ [19] with the catheter transformation:

7 $$\begin{aligned} _\textrm{s}^\textrm{4t}\pmb {T}= _\textrm{s}^0\pmb {T} \, _{0}^\textrm{4t}\pmb {T}(p,r,b,d) = \left[ \begin{array}{c|c} \pmb {R}^\textrm{s} & \pmb {P}^\textrm{s} \\ \hline {\textbf {0}}_{1\times 3} & 1 \end{array} \right] \end{aligned},$$

where $\pmb {P}^\textrm{s} \in \mathbb {R}^{3\times 1}$ and $\pmb {R}^\textrm{s} \in \mathbb {R}^{3\times 3}$ denote the position and orientation of the dock catheter tip with respect to the guide-sheath base frame $\{\textrm{F}^\textrm{s}\}$, respectively.

### Controller Design

In transseptal mitral interventions, the anatomical relationship between the fossa ovalis and the mitral leaflets (target) is generally similar across patients [40]. As a result, the dock catheter typically emerges from the guide sheath after crossing the fossa ovalis in a predictable initial configuration. However, patient-specific anatomical variations and procedural nuances still require fine positioning of the catheter tip. To manage this, we divide the control problem into two phases: an approach phase, for efficient navigation toward the mitral annulus, and a fine adjustment phase, for direct tip control near the valve.

#### Approaching Phase Using Joint-Space Control

In this phase, the dock catheter is advanced through the guide sheath (Fig. 1), and its tip is guided from the fossa ovalis toward a region near the mitral annulus. Initial motion is achieved through robotic translation, while subsequent movement can be preplanned based on a selected configuration, as anatomical structures in this region are relatively consistent across patients. The operator then drives the robot toward this configuration using joint-space control (Fig. 3a). This preselected configuration aims to position the catheter tip near the center of the mitral annulus, maximizing the available task space and simplifying the fine adjustment phase that follows.

**Fig. 4:**
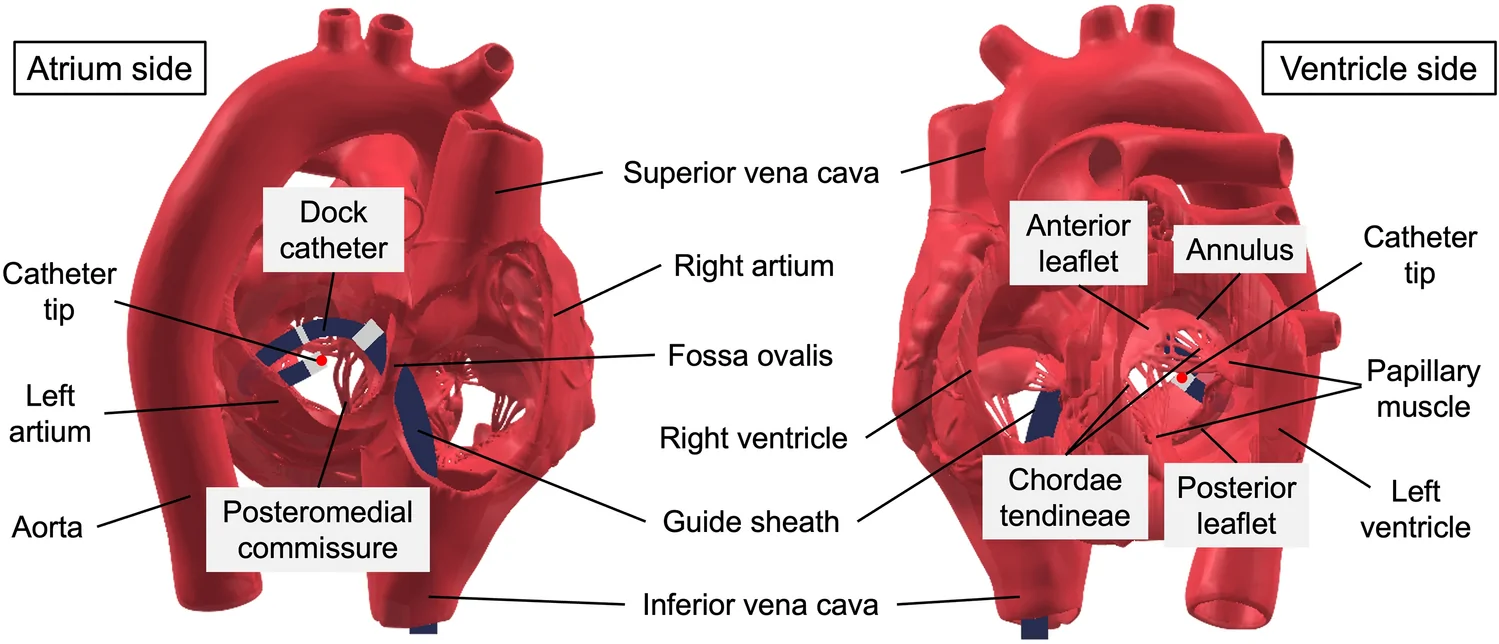
The approaching phase is demonstrated in a heart model with openings that reveal key anatomical structures. The goal is to position the dock catheter tip near the center of the mitral annulus. This is achieved by moving the catheter to a predefined configuration (10 mm, $-40^\circ$, $50^\circ$, $135^\circ$) using joint-space control

We assume a standard supine patient posture, as shown in Fig. 4. The left panel displays the heart from the atrial side, with openings in both the left and right atria to reveal the valve and the catheter. The right panel presents a view from the ventricular side, also with anatomical openings. The heart model is anatomically accurate and adapted from an echocardiography e-learning module by the University of Groningen (licensed under CC Attribution-NonCommercial-ShareAlike). The dock catheter is positioned within the heart, with its tip located near the center of the mitral annulus. This configuration could serve as the target, approximately represented by the values (10 mm, $-40^\circ$, $50^\circ$, $135^\circ$). High precision is not required at this stage; the goal is to reach the target region roughly, as fine adjustment will be performed in the second phase.

#### Fine Adjustment Phase Using Task-Space Control

**Fig. 5:**
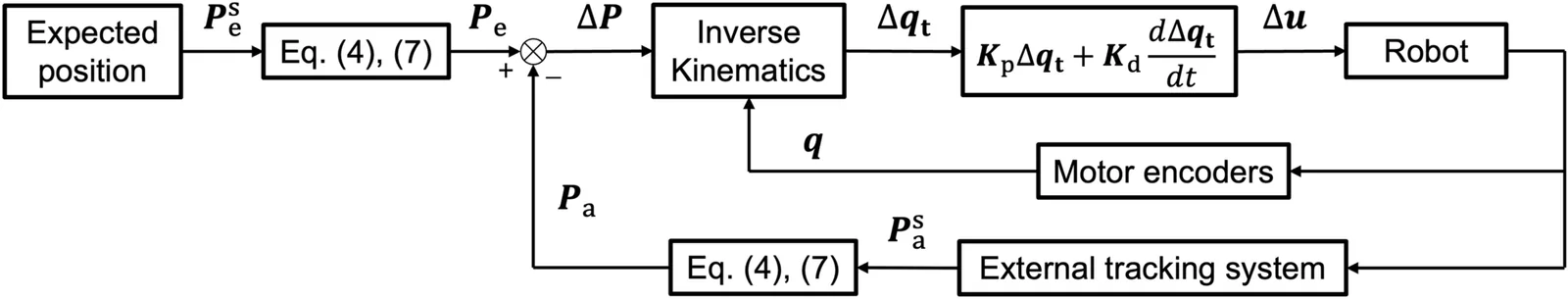
Control framework overview: The expected and measured tip positions are transformed into the dock catheter coordinate frame. Joint displacements are then computed using Jacobian-based inverse kinematics. Joint positions are incorporated as feedback to define a secondary objective for joint-limit avoidance. Finally, a PD controller regulates the control inputs

Once the catheter tip reaches the vicinity of the mitral annulus center, fine positioning becomes essential to ensure proper alignment and safe docking. To achieve this, a closed-loop task-space controller (shown in Fig. 5) is used to adjust the catheter tip’s position. The expected tip position in the guide-sheath coordinate frame is denoted as $\pmb {P}^\textrm{s}_\textrm{e}$, while the actual tip position, tracked by an external system, is denoted as $\pmb {P}^\textrm{s}_\textrm{a}$. Based on (4) and (7), both positions are transformed into $\{\textrm{F}^{0}\}$, the coordinate frame of the dock catheter, yielding $\pmb {P}_\textrm{e}$ and $\pmb {P}_\textrm{a}$. The position error is then computed as the difference between these two quantities, resulting in $\Delta \pmb {P}$.

Due to the complexity of the catheter tip’s forward kinematics, deriving an analytical inverse kinematics model is challenging. Consequently, a numerical solution is more suitable. To control the catheter tip position, we construct the Jacobian matrix $\pmb {J}$ (8), which defines the relationship between the tip position $\pmb {P}$ and the joint variables $\pmb {q}=[j_1,j_2,j_3,j_4]^\textrm{T}$. While the robot is kinematically redundant for tip position control, the joint variables have different physical units: the first joint is in millimeters, whereas the remaining three joints are in degrees. Then a diagonal scaling matrix $\pmb {S}$ is introduced to normalize the columns of the Jacobian, yielding the scaled Jacobian $\hat{\pmb {J}}$ as defined in (9).

8 $$\begin{aligned} \pmb {J} = \frac{\partial \pmb {P}}{\partial \pmb {q}} = \begin{bmatrix} \dfrac{\partial p_\textrm{x}}{\partial j_1}& \dfrac{\partial p_\textrm{x}}{\partial j_2}& \dfrac{\partial p_\textrm{x}}{\partial j_3}& \dfrac{\partial p_\textrm{x}}{\partial j_4}\\ \dfrac{\partial p_\textrm{y}}{\partial j_1}& \dfrac{\partial p_\textrm{y}}{\partial j_2}& \dfrac{\partial p_\textrm{y}}{\partial j_3}& \dfrac{\partial p_\textrm{y}}{\partial j_4}\\ \dfrac{\partial p_\textrm{z}}{\partial j_1}& \dfrac{\partial p_\textrm{z}}{\partial j_2}& \dfrac{\partial p_\textrm{z}}{\partial j_3}& \dfrac{\partial p_\textrm{z}}{\partial j_4} \end{bmatrix} \end{aligned}$$

9 $$\begin{aligned} \hat{\pmb {J}} = \pmb {J}\pmb {S} = \pmb {J}\,\textrm{diag}\left( \frac{1}{|\pmb {J}_{:,1}|}, \frac{1}{|\pmb {J}_{:,2}|}, \frac{1}{|\pmb {J}_{:,3}|}, \frac{1}{|\pmb {J}_{:,4}|} \right) \end{aligned},$$

where $\pmb {J}_{:,k}$ denotes the *k*-th column of the Jacobian matrix, and |·| represents the Euclidean (L2) norm.

To ensure stability near singularities, the damped pseudo-inverse of the Jacobian $\pmb {J}^{+}$ (10) is used to compute joint adjustments $\Delta \pmb {q}$ based on position errors $\Delta \pmb {P}$,

10 $$\begin{aligned} \begin{aligned} \Delta \pmb {q} = \pmb {J}^{+}\Delta \pmb {P} = \pmb {S} \hat{\pmb {J}}^\textrm{T} (\hat{\pmb {J}} \hat{\pmb {J}}^\textrm{T} + \lambda ^2 \pmb {I}_{3\times 3})^{-1}\Delta \pmb {P} \end{aligned} \end{aligned},$$

where λ and $\pmb {I}_{3\times 3}$ are the damping factor and identity matrix, respectively.

In addition, the joint adjustments $\Delta \pmb {q}$ computed from the Jacobian may cause the bending angle $b_3$ to exceed its valid range during iterative updates. To ensure physically feasible motion, constraints must be introduced to account for both mechanical and workspace limitations. A null-space optimization term is added to regulate joint motion without affecting the primary task. The null-space projector is defined using the original Jacobian as

11 $$\begin{aligned} \pmb {N} = \pmb {I}_{4\times 4} - \pmb {J}^{+}\pmb {J}. \end{aligned}$$

A secondary objective is then defined to enforce joint-limit avoidance for the third joint only. Specifically, a null-space motion vector $\pmb {z}$ is constructed as

12 $$\begin{aligned} \pmb {z} = \begin{bmatrix} 0&0&-k\left( j_3 - j_3^{\textrm{mid}}\right)&0 \end{bmatrix}^\textrm{T} \end{aligned},$$

where $j_3^{\textrm{mid}}$ is the midpoint of the allowable range of the third joint, and *k* is a positive gain. The final joint update that incorporates both the primary task and the secondary objective is then given by

13 $$\begin{aligned} \Delta \pmb {q}_\textrm{t} = \Delta \pmb {q} + \pmb {N}\pmb {z}. \end{aligned}$$

After computing the final joint adjustment $\Delta \pmb {q}_\textrm{t}$, a PD controller is applied to generate smooth and stable joint commands. The control law is

14 $$\begin{aligned} \Delta \pmb {u} = \pmb {K}_\textrm{p} \, \Delta \pmb {q}_\textrm{t} + \pmb {K}_\textrm{d} \, \displaystyle \frac{d \Delta \pmb {q}_\textrm{t}}{dt}, \end{aligned}$$

where $\pmb {K}_\textrm{p}$ and $\pmb {K}_\textrm{d}$ are the proportional and derivative gains, respectively, and $\Delta \pmb {u}$ represents the relative control signals sent to the motors. This PD loop provides the capability to guide the docking catheter toward the expected tip trajectory.

### Experimental Setup

To evaluate the performance of task-space control, we conducted catheter tip trajectory-following experiments. Besides this primary goal, the setup also helped identify potential hardware design issues and provided valuable data for future clinical studies.

**Fig. 6:**
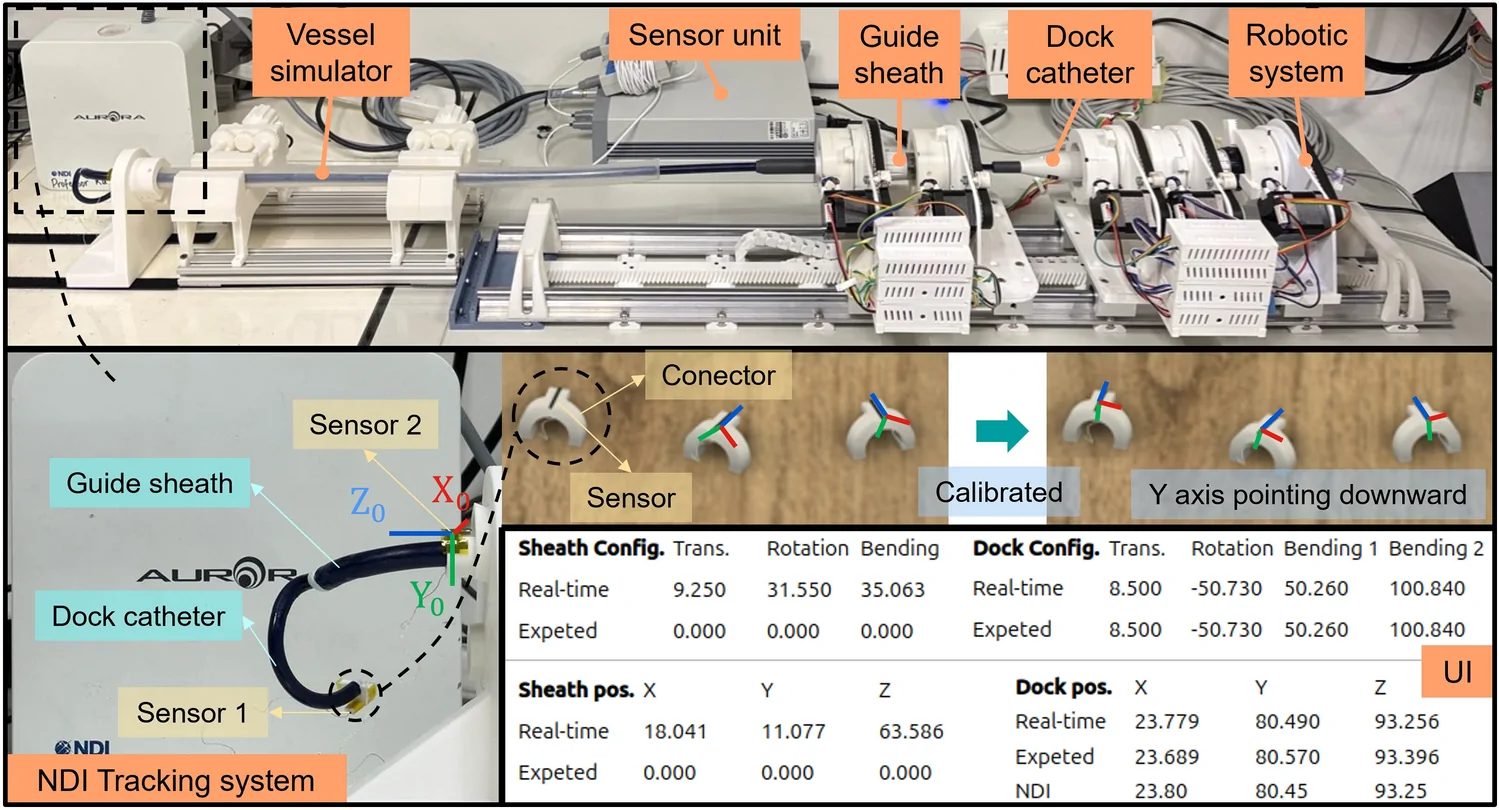
Experimental setup: The system includes the robotic platform (with guide sheath and dock catheter mounted), a vessel simulator, and a tracking system. The robot communicates with the UI via a CAN bus to execute control commands and display real-time feedback. The catheter is inserted through the vessel simulator to replicate the anatomical path from the femoral vein to the inferior vena cava. Two Aurora sensors are used to track catheter movement by measuring the positions of the sheath base and dock tip

The experimental setup is shown in Fig. 6. It includes the robotic system (with the guide sheath and dock catheter mounted in a concentric configuration, consistent with their clinical arrangement), a vessel simulator, and a tracking system. The sheath was inserted through the vessel simulator, which consists of a PVC tube secured by two vises, simulating the anatomical path from the femoral vein to the inferior vena cava. Clinically, once the sheath crosses the fossa ovalis, it is typically maintained in a relatively fixed position, with only minor adjustments. Accordingly, in this experiment, the sheath is treated as a structural constraint at the fossa ovalis, and the control accuracy of the dock catheter is evaluated. The task-space control algorithm has been implemented in the software platform with a control period of 80 ms. For the experimental procedure, the robot was first programmed to position the dock catheter in the configuration described in Section Approaching Phase Using Joint-Space Control. It was then executed to follow a predefined 20 × 10 mm$^2$ figure-eight (“8”) trajectory, consisting of five straight segments in the upper portion and a curved segment in the lower portion. The angles between adjacent straight segments are 45 and 90$^\circ$. Each straight segment was discretized into 200 evenly spaced points, while the curved segment was discretized into 1000 points. The catheter motion starts from the center point, and the trajectory is oriented to ensure three-dimensional validation. Catheter movement was tracked using the NDI Aurora electromagnetic tracking system (Northern Digital Inc., Waterloo, Ontario, Canada). Two 6-DOF sensors were used to measure the positions and orientations of the sheath base and dock catheter tip within the field generator. The nominal tracking accuracy of the system is approximately 0.48 mm root mean square (RMS) in position and $0.30^\circ$ in orientation [41]. Because the sensor was attached to the connector with an unknown orientation, we placed the connector flat on a table and recorded 20 poses in 20 s. From this dataset, we calculated the orientation angles that aligned the Y-axis perpendicular to the horizontal plane (pointing downward toward the table). This average orientation was defined as the offset angle. The actual tip pose was then computed by applying this offset and calculating the transformation from Sensor 1 to Sensor 2. The pose is thus plotted in the coordinate system $\{\textrm{F}^\textrm{s}\}$.

The NDI data stream was integrated into the software platform, allowing both the catheter tip position from the NDI Aurora and the corresponding reference (expected) position to be acquired within the same interface. Both data streams were sampled at a fixed rate of 40 Hz, ensuring that each recorded pair of measurements shared the same timestamp. Therefore, the data were inherently synchronized, and no additional temporal alignment was required. Error analysis was performed by comparing the NDI tip position with the expected tip position. The tracking error was quantified as the Euclidean distance between the estimated and reference positions. The performance was evaluated using the maximum error and RMS error across the trajectory. This procedure was repeated three times.

## Results

**Fig. 7:**
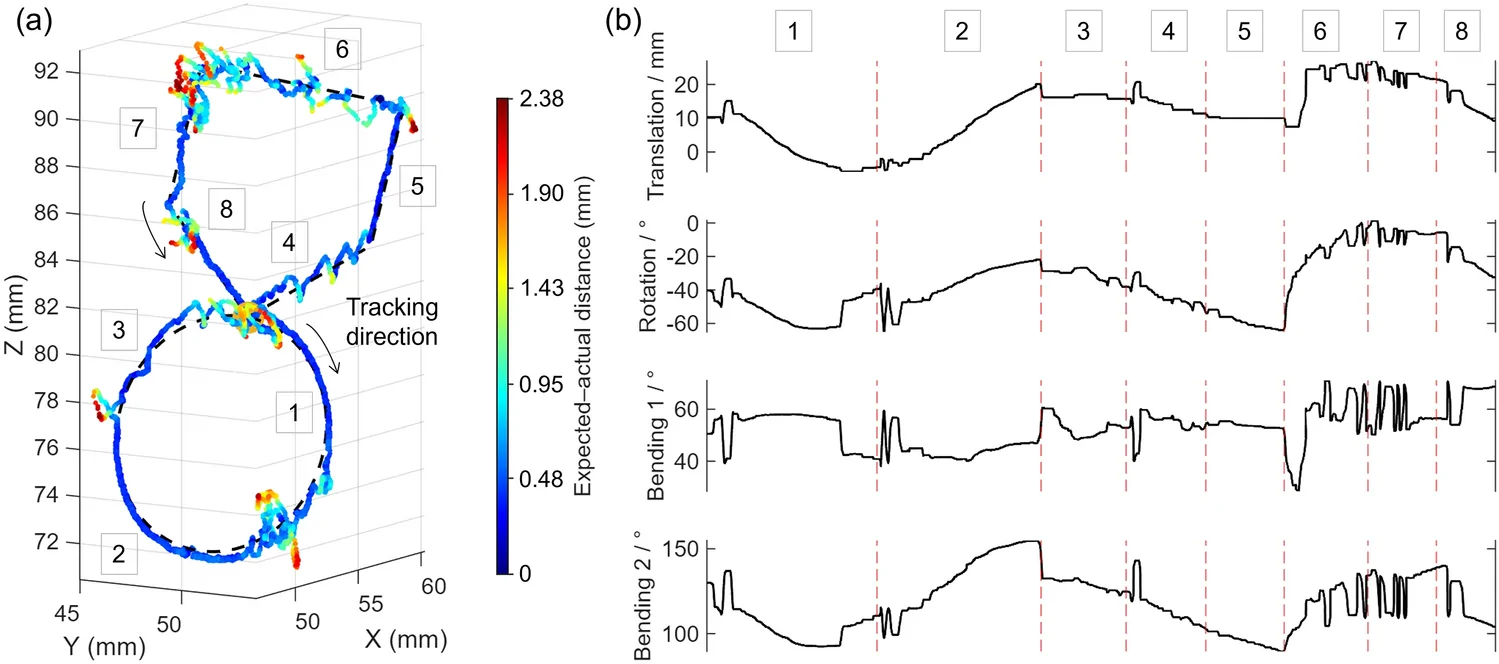
Catheter tip following results:** a** Actual tip positions (colored markers) compared with the expected trajectory (black dashed lines). The color transitions from blue to red with increasing error. The path is divided into eight segments for analysis.** b** DoF values during trajectory tracking, with segments separated by red dashed lines.

The parameters in (10), (12), and (14) are set as follows: λ=0.1, k=0.2, $\pmb {K}_\textrm{p}$ = diag(0.10, 0.15, 0.20, 0.20), and $\pmb {K}_\textrm{d}$ = diag(0.008, 0.012, 0.016, 0.016). The position error for trajectory tracking from one representative trial is shown in Fig. 7a. The solid black line represents the expected trajectory, while the colored markers indicate the actual catheter tip trajectory, with colors transitioning from blue to red as the distance increases. The values for each DoF are shown in Fig. 7b. The actual trajectory in Fig. 7a is divided into eight segments based on error values and trajectory patterns, with the numbers shown in squares for reference. The DoF values corresponding to each segment are separated by red dashed lines in Fig. 7b.

The catheter tip begins at the center of the trajectory. As the tip moves into Segment 1, the catheter vibrates, which is reflected in the oscillations of the DoF values, and the error rises to approximately 2 mm. This is likely due to hysteresis and the time delay associated with catheter deformation. The tip then follows the right circular path along the +X, +Y, and -Z directions. The tracking error remains low, with predominantly blue markers and an average of about 0.45 mm, which is close to the nominal accuracy of the sensing system. This indicates good initialization and demonstrates the best achievable tracking performance under the current sensing precision. When the motion transitions from Segment 1 to Segment 2 (direction change from +X to -X), a noticeable increase in error occurs, with peaks reaching approximately 2.10 mm. This reflects a transient response with oscillation, likely caused by control lag, model inaccuracies, and the sudden change in motion direction. In Segment 2, along the left-lower circular path, the oscillations gradually settle and the error stabilizes. However, during the transition from Segment 2 to Segment 3 (from -X back to +X), similar oscillatory behavior reappears, with the error remaining around 1.2 mm. This transition settles more quickly and occurs as the trajectory crosses the center. The oscillation persists into Segment 4, likely due to a slight upward change in direction. The system does not have sufficient time to fully damp the previous oscillations, and the error remains around 1.3 mm. In Segment 5, where the motion is primarily along the +Z direction, the error decreases significantly to approximately 0.50 mm. Rotation and bending 2 are active, while translation and bending 1 remain relatively stable. The transition between Segments 5 and 6 involves a 90$^\circ$ change in direction, causing abrupt changes in the DoF values. In Segment 6, the error increases slightly to the range of 1.2 to 1.6 mm, indicating higher coordination demands among the joints. The largest error occurs during the transition from Segment 6 to Segment 7, reaching up to 2.38 mm. This transition involves a significant change in direction, which adds to the existing error and increases it from about 1.2 mm to 2.38 mm. The system also takes longer to settle, highlighting limitations in dynamic response and coordination. Finally, in Segment 8, as the tip returns to the center, the error decreases again to a relatively low level. A summary of all results is presented in Table 1. Similar patterns are observed in the other two trials, with larger errors consistently occurring at transition regions. The maximum errors are 2.53 mm and 2.46 mm, respectively, indicating consistent performance across trials. Overall, this demonstrates the coordination of all four DoFs in tracking a trajectory with varying motion patterns. The results indicate that the controller performs well after settling but is challenged by rapid directional changes that require coordinated multi joint actuation. The observed error peaks are mainly attributed to dynamic limitations, model mismatch, and coupling effects between joints.

**Table 1:** Positional error analysis across three trials

Errors (mm)	Maximum	RMS	SD
Trial 1	2.38	0.54	0.26
Trial 2	2.53	0.45	0.20
Trial 3	2.46	0.43	0.17

## Discussion

In this study, we present the design of MitraNav, a robotic system developed for dock-based TMVR, along with the modeling and task-space control of the dock catheter. Building on our previously validated guide-sheath robotic platform [19], MitraNav upgrades the system to support concentric, coordinated manipulation of both the guide sheath and a functional catheter. This capability is important in procedures where the guide sheath is required to compensate for route offsets [42, 43]. Similar concepts have been adopted in commercial systems, such as the Magellan Robotic Catheter System (Hansen Medical, Mountain View, California, USA) [44]. MitraNav can manipulate both the balloon catheter and the dock catheter, a versatility that is valuable because TMVR procedures may require different delivery catheters. This flexibility improves clinical practicality and increases the likelihood of adoption in catheter laboratories. Some studies have applied a similar concept to develop robotic systems capable of manipulating multiple ablation catheters [22, 45].

Compared with existing robotics systems for mitral interventions [27–35], this is the first system designed specifically for dock-based TMVR. Its key distinctions are the ability to manipulate both balloon and dock catheters, as described above, and the task-space control of a two-segment catheter for docking. From a technical perspective, the dock catheter can be classified as a two-segment continuum body. Building on our previous work addressing catheter nonlinearities such as hysteresis and dead zones [36], we constructed the kinematic model used in this study. Trajectory-following experiments demonstrate feasibility of direct tip control, with maximum errors of 2.38 mm, 2.53 mm, and 2.46 mm in three trials. These positional errors are within the range of acceptable accuracy commonly considered for catheter-based cardiac interventions, where navigation and localization errors on the order of  1.5−3.0 mm are reported in structural heart procedures guided by imaging and navigation systems [46]. Moreover, because the dock catheter is repositionable before release, small errors can be corrected under visual feedback.

Despite these advantages, several limitations remain. First, the current prototype requires manual reconfiguration when switching catheters, as the catheter must be inserted into the actuation module. A redesigned cartridge-style module could allow cardiologists to replace catheters more quickly by simply opening and closing a housing mechanism. Second, control accuracy could be improved by incorporating real-time intraoperative imaging, such as fluoroscopy or echocardiography [47, 48]. This approach would not require additional sensors, as it would rely directly on imaging already acquired during the procedure. Finally, validation has thus been limited to bench testing and has not tested the influence of chordal entanglement or failure to adequately encircle the subvalvular apparatus [20]. Further work should include experiments in in-vitro heart models and, ultimately, in-vivo studies to evaluate performance under physiological forces, tissue interaction, and dynamic cardiac motion.

In conclusion, this work presents the first robotic system specifically designed for dock-based TMVR, demonstrating a unified platform capable of manipulating multiple types of catheters. It also investigates two-segment continuum control in the context of mitral interventions, providing a foundation for standardized, image-guided, and robot-assisted valve procedures. This approach has the potential to reduce operator dependency, shorten the learning curve for less-experienced users, and enable future semi-autonomous workflows in structural heart interventions. Future work will focus on four main directions: (1) Mechanism refinement: improving the mechanisms to enhance usability and to satisfy clinical sterilization and disinfection requirements. (2) Complete workflow control: extending the current framework to validate the guide sheath–commander catheter combination, enabling a complete procedural workflow. (3) Imaging feedback integration: integrating imaging feedback to simplify the feedback system and further improve control accuracy. (4) Clinical validation: conducting systematic studies in procedural environments to evaluate feasibility, safety, and effectiveness.
